# Efficacy of Intraperitoneal Administration of PEGylated NELL-1 for Bone Formation

**DOI:** 10.1089/biores.2016.0018

**Published:** 2016-06-01

**Authors:** Justine Tanjaya, Yulong Zhang, Soonchul Lee, Jiayu Shi, Eric Chen, Pia Ang, Xinli Zhang, Sotirios Tetradis, Kang Ting, Benjamin Wu, Chia Soo, Jin Hee Kwak

**Affiliations:** ^1^ Division of Growth and Development and the Section of Orthodontics, School of Dentistry, University of California, Los Angeles, Los Angeles, California.; ^2^ Department of Bioengineering, University of California, Los Angeles, Los Angeles, California.; ^3^ Department of Materials Science and Engineering, University of California, Los Angeles, Los Angeles, California.; ^4^ Department of Orthopaedic Surgery, CHA Bundang Medical Center, CHA University, Pocheon, South Korea.; ^5^ Department of Orthopaedic Surgery and the Orthopaedic Hospital Research Center, University of California, Los Angeles, Los Angeles, California.; ^6^ Section of Oral and Maxillofacial Radiology, School of Dentistry, University of California, Los Angeles, Los Angeles, California.; ^7^ Division of Plastic and Reconstructive Surgery, Department of Surgery, David Geffen School of Medicine, University of California, Los Angeles, Los Angeles, California.

**Keywords:** biodistribution, bone formation, intraperitoneal administration, osteoporosis, PEGylated NELL-1

## Abstract

Systemically delivered NEL-like molecule-1 (NELL-1), a potent pro-osteogenic protein, promotes bone formation in healthy and osteoporotic mouse models. PEGylation of NELL-1 (NELL-PEG) increases the half-life of the protein in a mouse model without compromising its osteogenic potential, thereby improving its pharmacokinetics upon systemic delivery. This study consists of a twofold approach: a biodistribution test and an *in vivo* osteogenic potential test. The biodistribution test compared two commonly used administration methods for drug delivery other than intravenous—intraperitoneal (IP) and subcutaneous (SC)—to examine NELL-PEG biodistribution in mice. Compared to a single-dose SC injection (1.25 mg/kg), a single-dose IP administration yielded a higher protein uptake in the targeted bone sites. When the IP injection dose was doubled to 2.5 mg/kg, the protein remained in the femurs, tibias, and vertebrae for up to 72 h. Next, based on the results of the biodistribution study, IP administration was selected to further investigate the *in vivo* osteogenic effects of weekly NELL-PEG injection (q7d). *In vivo*, the IP administered NELL-PEG group showed significantly greater bone mineral density, bone volume fraction, and trabecular bone formation in the targeted bone sites compared to the phosphate-buffered saline control. In summary, weekly NELL-PEG injection via IP administration successfully enhanced the overall bone quality. These findings demonstrate that systemic delivery of NELL-PEG via IP administration may serve as an effective osteogenic therapy for preventing and treating osteoporosis.

## Introduction

Human NEL-like molecule-1 (NELL-1), a potent growth factor that is highly specific to the osteochondral lineage, was first identified by its overexpression in the context of human unilateral craniosynostosis (UCS), a congenital cranial defect characterized by premature fusion of one of the sutures in the developing cranium.^1,2^ Over the past two decades, NELL-1 was closely studied for its local bone formation effects^3–9^; more recently, NELL-1 has demonstrated its osteogenic potential as a systemic therapy.^10–12^ Mechanistically, NELL-1 affects multiple signaling pathways and has the potential to differentiate the multipotent bone mesenchymal stem cells (BMSCs) into osteoblasts by acting specifically through the Runx2 and canonical Wnt signaling pathway and activating the ERK/JNK/MAPK pathway.^2,11–13^ Simultaneously, NELL-1 suppresses adipogenesis through the peroxisome proliferator-activated receptor gamma (PPARγ) and CCAAT/enhancer-binding protein alpha (C/EBPα) pathways.^14^

Osteoporosis is a prevalent metabolic disease that affects more than 200 million people worldwide.^15–17^ Existing osteoporosis therapeutic agents fall into two classes: (i) antiresorptives, such as bisphosphonates, which slow down bone resorption, and (ii) anabolic agents, such as teriparatide parathyroid hormone (PTH 1–34 and PTH 1–84), the only Food and Drug Administration (FDA)-approved anabolic treatment agent for osteoporosis, which targets the stimulation of osteoblast-mediated bone formation.^18–22^ Bisphosphonates represent the first line of treatment for osteoporosis^23^; however, they are associated with osteonecrosis of the jaw and common side effects, such as esophageal irritation and gastrointestinal discomfort, and even transient flu-like symptoms lead to up to 20% of those who are taking the drug to discontinue it.^24–27^ New therapeutics, such as odanacatib, one of the few cathepsin K inhibitors that showed adequate efficiency and safety,^28^ was recently reported in various clinical trials to increase fracture risk.^23,29,30^ In addition, denosumab (an antiosteoclastic agent) can induce hypocalcemia in patients with severe renal impairment,^25^ while prolonged antisclerostin (anti-Wnt inhibitor) treatment has prompted concerns about cardiovascular health and safety.^23,31^ Therefore, there is a pressing need to develop new therapies for the treatment of osteoporosis that are not only anabolic and antiosteoclastic but also have fewer safety concerns.

A recent genome-wide association study identified *NELL-1* polymorphisms in patients with reduced bone mineral density (BMD), suggesting that *NELL-1* gene polymorphisms are associated with osteoporosis.^32^ NELL-1 also has demonstrated the ability to increase BMSC numbers, promote osteogenesis, and suppress osteoclastic activity and adipogenesis, with fewer adverse effects compared to existing therapies.^2,14,28,33–37^ When an ovariectomized (OVX) rat model was used to mimic the human osteoporotic bone loss, local delivery of NELL-1 into the femoral intramedullary cavities enhanced the bone quality and successfully prevented osteoporosis-induced bone loss.^6^ Similarly, systemic delivery of rNELL-1 via intravenous (IV) administration demonstrated significant bone augmentation in osteoporosis-induced mice.^12^ Since osteoporosis is a systemic skeletal disorder, it is crucial for therapeutic agents to be administered systemically to enhance the overall bone quality. Notwithstanding the proven efficacy of NELL-1 to prevent bone loss, the clinical use of systemic rNELL-1 therapy was deemed to be quite limited due to the burden of an every other day (q2d) administration schedule.^12^

PEGylation is an FDA-approved method of modifying biological molecules of a protein using covalent conjugation of polyethylene glycol (PEG) molecule drug.^38–40^ Recently, our group has established that PEGylated NELL-1 (NELL-PEG) demonstrates higher thermal stability and prolongs systemic circulation by preserving the osteogenic effects of NELL without any considerable cytotoxicity.^10^ The applicability and safety of NELL-PEG were further examined in an *in vivo* study, where its weekly systemic administration through IV tail injection resulted in increased BMD, greater bone trabecular formation, and reduced bone resorption in the targeted bone sites.^11^

The aforementioned studies of NELL-PEG via the IV route have successfully demonstrated the anabolic and antiresorptive functions of the protein by promoting bone formation and reversing bone loss without undue adverse effect of immunocytotoxicity.^10,11^ However, further optimization of the therapy to allow intraperitoneal (IP) or subcutaneous (SC) administration was called for to develop it into a safer and patient-friendly therapy. Given the benefits of greater volume administration and reduced irritation to the veins, IP and SC injections are frequently reported to be as effective as IV injection and may be preferable to IV injection.^41–45^ To test our hypothesis that systemic NELL-PEG therapy delivered via the IP or SC route could prevent and treat osteoporosis comparable to that via the IV route, in the present study, we first compared the protein distribution of the IP and SC NELL-PEG administration methods. Next, we examined the efficacy of weekly IP NELL-PEG administration in promoting bone formation and reversing bone loss. Furthermore, an *in vivo* mouse model was used to investigate the osteogenic potential of weekly NELL-PEG injection via the IP route.

## Materials and Methods

### Animals

Three-month-old female CD-1 and C57BL/6J mice were obtained from Charles River Laboratories and maintained under standard conditions under the supervision of the Division of Laboratory Animal Medicine (DLAM) at the UCLA. Animals were housed individually per cage and maintained on a 12-h light–12-h dark cycle with *ad libitum* access to laboratory rodent chow and water. The animal protocol was approved by the Office of Animal Research Oversight (OARO) and the Chancellor's Animal Research Committee (ARC) at the UCLA.

### Biodistribution study

To investigate the biodistribution of NELL-PEG protein for various administration methods, nine female CD-1 adult mice were randomly divided into three groups (one group of NELL-PEG injection via IP administration, one group of NELL-PEG injection via SC injection, and one phosphate-buffered saline [PBS] control group via IP administration). For the first part of the biodistribution study, animals were either subjected to 100 μL of NELL-PEG solution via IP injection (1.25 mg/kg) and NELL-PEG solution via SC injection (1.25 mg/kg) or assigned to the control group with PBS solution injection. The second part of the biodistribution study was performed to compare the protein distribution of NELL-PEG injection via IP administration at two different time points (48 and 72 h) postinjection. Nine female CD-1 adult mice were randomly divided into three groups (two groups of NELL-PEG injection via IP administration and one PBS control group). Animals were either administered with 100 μL of NELL-PEG solution via IP injection (2 × dose of 2.5 mg/kg) or assigned to the control group with PBS solution injection. The first group of NELL-PEG-treated animals was sacrificed at 48 h postinjection, while the second group was sacrificed at 72 h. The dose was calculated based on the protein content. NELL-PEG was labeled with VivoTag 680XL (PerkinElmer). At 48 and 72 h postinjection, all mice were euthanized, and the organs (liver, kidney, spleen, heart, lungs, brain, muscle, fat, ovary, calvaria, vertebrae, femur, and tibia) were harvested, weighed, and imaged with the IVIS Lumina II optical imaging system (Caliper Life Sciences). Quantification of the total amount of protein uptake by 1 g tissue weight of the organs was calculated and plotted.

### *In vivo* assessment of BMD by dual-energy X-ray absorptiometry

Fourteen female C57BL/6J adult mice were randomly divided into the NELL-PEG group and the PBS control group and injected with either NELL-PEG (2.5 mg/kg) or PBS intraperitoneally every 7 days. Subsequently, changes in BMD were monitored every 2 weeks with dual-energy X-ray absorptiometry (DXA; PIXImus2; GE Lunar Corp.). Longitudinal assessment of the whole body (excluding head), distal femur, and lumbar vertebrae BMD (g/cm^2^) was performed every 2 weeks starting at the baseline until the end of the study.

### *In vivo* assessment of bone turnover rate by live micro-positron emission tomography/computed tomography scanning with ^18^F-NaF ion

Before injection, all animals were warmed on a heating pad for 15 min. Afterward, mice were injected with an average of 77.5 μCi of ^18^F-NaF ion via tail vein injection using a tuberculin syringe and maintained under anesthesia (2% isofluorane) on a heated induction chamber during the 1-h tracer uptake. All animals underwent micro-positron emission tomography (micro-PET) scanning (Siemens Medical Solutions, Inc.), followed by micro-computed tomography (micro-CT) scanning (Siemens Medical Solutions, Inc.) with a 10-min acquisition time for both scans. Plain anteroposterior radiographs (micro-CT) were superimposed on reconstructed PET images using A Medical Image Data Examiner (AMIDE) software version 0.7.15. Mean signal intensity (%ID/cc) within the volume of interest was calculated using the AMIDE data analysis tool. Values were then corrected for the actual tracer injected dose. Rendered three-dimensional images were generated by AMIDE, and a %ID/cc threshold of 80/3 was used.

### *Ex vivo* assessment of bone architecture by micro-CT

At the final time point, all mice were euthanized in a CO_2_ chamber with the appropriate CO_2_ concentrations and exposure times. Concomitantly, all organs were harvested, cleaned of soft tissue, and then stored in 4% paraformaldehyde (PFA) at −4°C. A total of 28 femurs (both hind limbs) and 14 vertebrae were scanned with SkyScan 1172 (Bruker microCT N.V.). For distal femoral analyses, the total length was ∼2.5 mm, with an offset of 1.5 mm to the growth plate. For lower lumbar vertebrae, transverse micro-CT slices were acquired for the entire vertebral body, and trabecular bone was evaluated within the region of 0.3 mm away from the growth plate. For trabecular morphology, assessment of bone volume fraction (BV/TV, %), trabecular thickness (Tb. Th, mm), trabecular number (Tb. N, mm^−1^), and trabecular separation (Tb. Sp, mm) were used. All analyses were performed with CTAn software (Bruker microCT N.V.).

### Histology and quantitative histomorphometry

All right femurs were put in a 19% EDTA solution for 14 days, and the solution was changed daily. Subsequently, all samples were sent to the Translational Pathology Core Laboratory (TPCL) at the UCLA Department of Pathology for paraffin embedding. Longitudinal sections of 5 μm thickness were created by microtome. All slides were used for hematoxylin and eosin (H&E), trichrome, tartrate-resistant acid phosphatase (TRAP), and osteocalcin (OCN) staining. All specimens were analyzed under an Olympus BX51 microscope (Olympus Corp.) using cellSens software version 1.6 (Olympus Corp.). Six consecutive images at the distal femur region were acquired for OCN and TRAP analyses, which were completed by three blinded examiners using ImageJ software v1.48 (National Institutes of Health). Parameters of osteocalcin+bone-lining cells per bone perimeter (OCN+cells/Bpm, mm^−1^) and surface (Ob.S/Bs, %), TRAP+bone-lining cells per bone perimeter (TRAP+cells/Bpm, mm^−1^), and surface (Oc.S/Bs, %) were used as previously reported.^12^

### Statistical analysis

Standard descriptive statistics and 95% confidence intervals were estimated, and the distributions of the parameters were assessed for normal distribution. For longitudinal data, percent change in each parameter over time was estimated using a linear mixed model. Independent sample *t*-tests were used to compare means between the NELL-PEG-treated group and the PBS control group. Data are presented as mean ± standard error of the mean, with *p < 0.05 and **p < 0.01.

## Results

### Biodistribution study

**FIG. 1.**
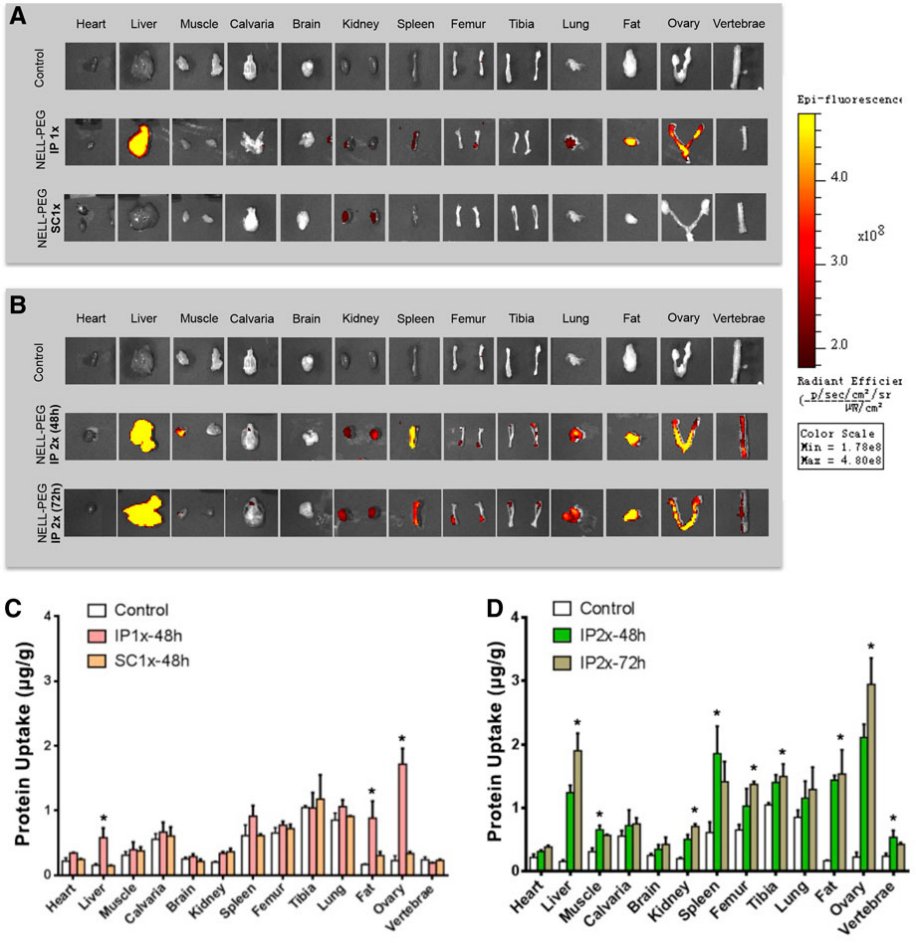
Biodistribution study was performed to compare the protein distribution of NELL-PEG labeled with VivoTag 680XL via the IP and SC administration. **(A)** The IP and SC routes were compared after CD-1 mice were subjected to a single dose 1 × (1.25 mg/kg) of NELL-PEG. *Ex vivo* images of the organs were collected at 48 h postinjection. The IP injection group showed detectable protein retention in the organs of liver, spleen, kidney, lung, fat, ovary, and femur; however, a similar finding was not observed in the SC injection group. **(B)** A double dose 2 × (2.5 mg/kg) of NELL-PEG was administered via the IP route, and the organs were dissected and imaged at two different time points (48 and 72 h) postinjection. **(C)** Quantification of the amount of protein distributed to different organs (μg/g). The biodistribution study confirmed that a single-dose injection of NELL-PEG via IP administration has significantly higher protein uptake in the liver, fat, and ovary compared with the PBS control group. **(D)** A double-dose injection of NELL-PEG via the IP administration showed that the quantification of the images at 48 h postinjection has significantly higher protein uptake in the targeted bone tissues, namely the femur, tibia, and vertebrae, compared with the control group. Quantification of the protein uptake at 72 h postinjection revealed a higher amount of protein in the liver and kidney compared with that at 48 h postinjection. *Significant difference (*p* < 0.05) between treatment and control group means. Error bars represent standard deviation. IP, intraperitoneal; NELL-PEG, PEGylation of NELL-1; PBS, phosphate-buffered saline; SC, subcutaneous.

The biodistribution study was performed to compare the distribution of protein across various administration routes of NELL-PEG labeled with VivoTag 680XL. The first part of the study compared a single dose (1.25 mg/kg) of NELL-PEG injection via the IP and SC injection routes (Fig. 1A, C). *Ex vivo* fluorescence images at 48 h postinjection showed a high hepatic uptake of the protein for a single-dose IP injection of NELL-PEG, suggesting that the protein was absorbed and highly metabolized by the liver. Other organs and tissues, such as the spleen, kidney, lung, fat, ovary, and femur, also exhibited some protein retention (Fig. 1A). The fat and ovary also revealed high uptake due to their location near the injection site (Fig. 1A). The protein was not distributed to the liver via the SC injection route, and there was no significant difference compared with the PBS control group (Fig. 1A, C). Thus, the IP injection route was selected to further test the *in vivo* osteogenic potential of NELL-PEG with a double-dose injection (2.5 mg/kg). To further examine the protein distribution of a double-dose NELL-PEG injection via the IP route, the organs were harvested and imaged at two time points: 48 and 72 h (Fig. 1B, D). At 48 h postinjection, targeted bone tissues, such as the femurs, tibias, and vertebrae, exhibited a great amount of retention. In contrast, images at 72 h exhibited a greater amount of NELL-PEG in the liver and kidney compared with those at 48 h, suggesting not only a greater amount of protein was metabolized over a longer period but also more protein was distributed to the overall organs (Fig. 1D).

### BMD by DXA

**FIG. 2.**
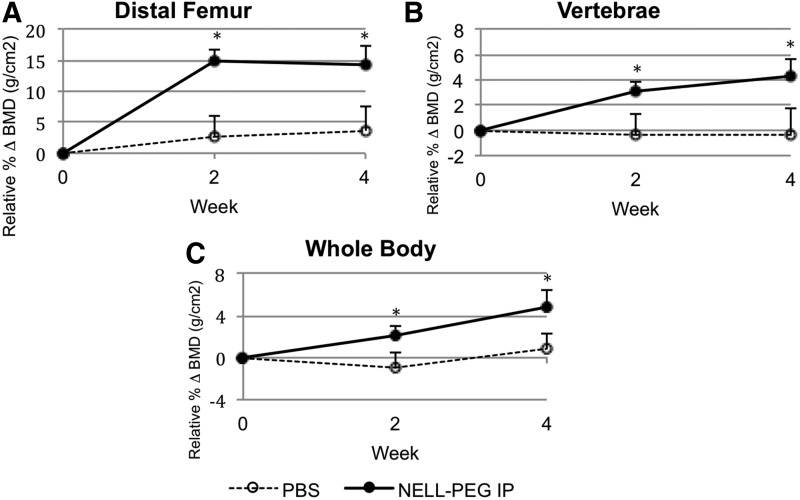
To monitor the changes in BMD, DXA scan was performed. Presented are mean changes in trabecular BMD at the second and fourth weeks of treatment for NELL-PEG-treated group and PBS control group at distal femoral metaphysis **(A)**, lower lumbar vertebral body **(B)**, and the whole body **(C)**. NELL-PEG-treated group is represented by solid line, whereas PBS control group is shown as dashed line. Compared with the control, NELL-PEG group shows significantly greater BMD increments relative to the week 0 baseline, and it gradually increases until the end of the treatment. *Significant difference (*p* < 0.05) between treatment and control group means. Error bars represent standard error of the mean. BMD, bone mineral density; DXA, dual-energy X-ray absorptiometry.

To dynamically monitor BMD, DXA scans were performed throughout the study (Fig. 2). Weekly administration of NELL-PEG via the IP route revealed a significant increase of BMD in the distal femur beginning from the second week of treatment. By the fourth week, the relative BMD increased by 14.27% compared to week 0 baseline and then plateaued at a level significantly higher than that of the PBS control group (Fig. 2A). The increase in vertebral BMD followed a different pattern than that of the femoral BMD, sustaining a gradual increase up to 4.25% until the end of the treatment (Fig. 2B). Total BMD increased rapidly at each time point during treatment; meanwhile, the total BMD of the control group remained the same (Fig. 2C).

### Bone turnover rate by live micro-PET/CT scanning with ^18^F-NaF ion

**FIG. 3.**
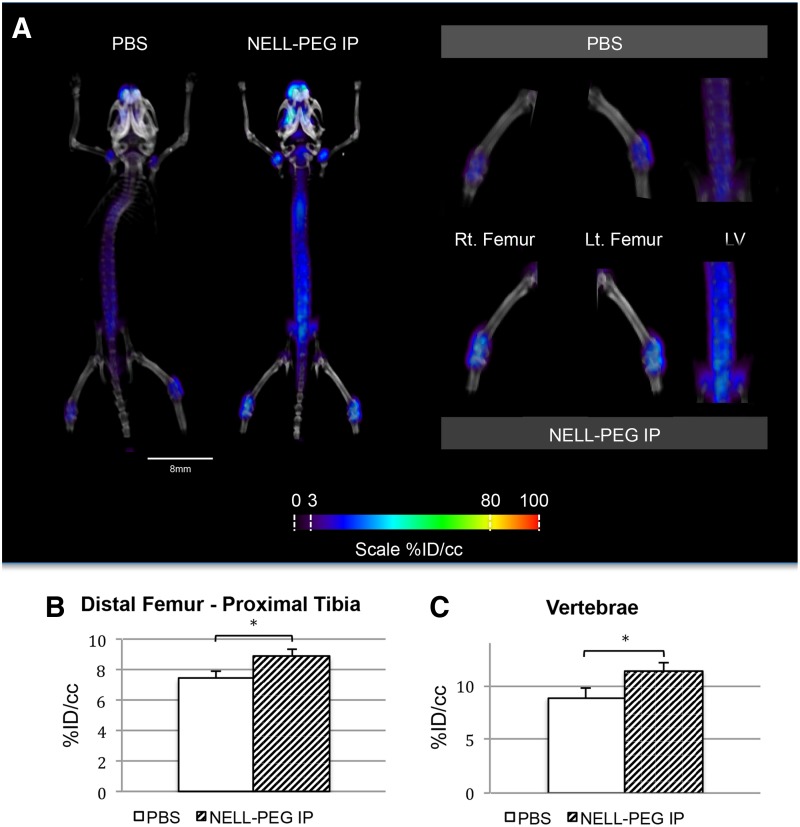
**(A)** Representative live micro-PET/CT images of NELL-PEG-treated group revealed a higher uptake of ^18^F-NaF ion over time, which corresponds to increased bone turnover rate in the targeted bone tissues, particularly the proximal humeri, vertebral body, distal femur, and proximal tibia compared with the PBS control group. ROIs were drawn at the distal femur–proximal tibia region and the lower lumbar region to encompass the areas that show high signal intensity. **(B, C)** Quantification of mean value at the distal femur–proximal tibia region and lower lumbar region (%ID/cc) at the fourth week postinjection. NELL-PEG-treated group exhibited significantly greater concentration of ^18^F-NaF ion uptake compared with the PBS control group. *Significant difference (*p* < 0.05) compared with the control group. Error bars represent standard error of the mean. Micro-PET/CT, micro-positron emission tomography/computed tomography; ROIs, regions of interest.

Tracer uptake of ^18^F-NaF was measured from the micro-PET/CT scans to assess the bone turnover rate. The results demonstrated that weekly injection of NELL-PEG increased the net uptake of ^18^F-NaF ions at the final time point (Fig. 3). Overall, the micro-PET scans revealed increased signal intensity in the calvaria, the axial skeleton (thoracic and lumbar vertebrae), and around the growth plates of the appendicular bones, such as the proximal humeri, distal femurs, and proximal tibia (Fig. 3A). Quantification of the mean value ratio at the distal femur–proximal tibia region of the treatment group exhibited significantly higher uptake of ^18^F-NaF tracer in comparison to the control group (Fig. 3B). The lower lumbar vertebrae also revealed high signal intensity, with statistically significant differences between the two groups (Fig. 3C).

### Bone architecture by micro-CT

**FIG. 4.**
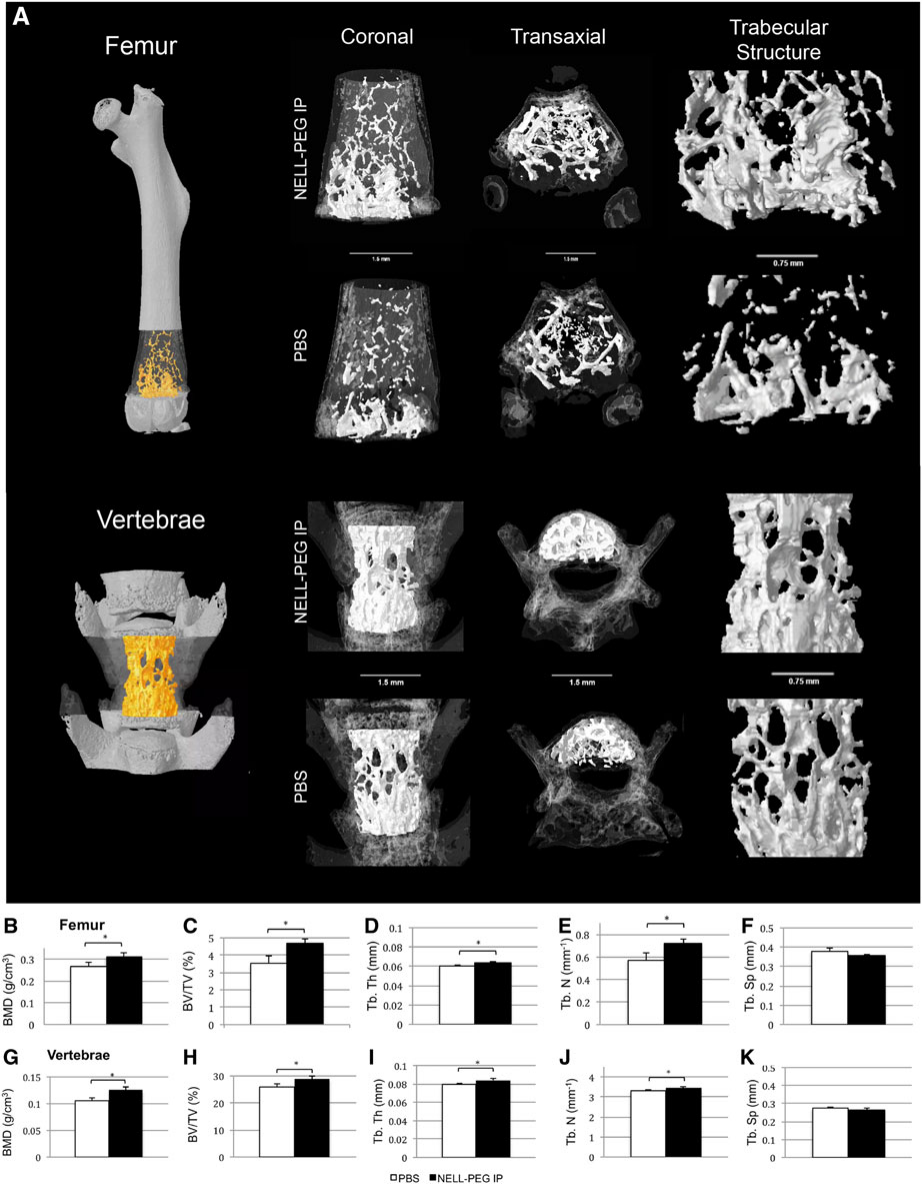
*Ex vivo* micro-CT results at the fourth week posttreatment. **(A)** Representative three-dimensional volume rendered micro-CT images showing comparison of trabecular bone architecture at the distal femoral metaphysis and the lumbar vertebrae column in coronal and transaxial views. **(A**, rightmost column**)** Trabecular structure was magnified from each representative sample. **(B–K)** Trabecular bone architecture assessment by micro-CT for BMD, BV/TV, Tb. Th, Tb. N, and Tb. Sp. NELL-PEG group shows significant increases of BMD, BV/TV, and improvement in trabecular structures at the distal femoral metaphysis and vertebral column compared with the PBS control group. *Significant difference (*p* < 0.05) compared with the control group. Error bars represent standard error of the mean. BV/TV, bone volume fraction.

Comparisons of trabecular bone architecture at the distal femoral metaphysis region and the lower lumbar region between the NELL-PEG injection group and the PBS control group are shown in Figure 4. In the NELL-PEG group, robust trabecular bone formation was observed at the distal femoral metaphysis region (Fig. 4A). BMD was significantly greater in the NELL-PEG group (Fig. 4B, G), which was consistent with our hind limb and vertebrae DXA analyses. The trabecular morphology assessment demonstrated a statistically significant difference in BV/TV between the NELL-PEG group and the PBS control, indicating that there was a great amount of bone augmentation within the bone volume at the femurs and the vertebral body (Fig. 4C, H). The trabecular bone architecture at the lower lumbar vertebrae exhibited a significantly higher trabecular thickness and number compared with the control (Fig. 4I). Similarly, the trabecular architecture at the distal femurs also exhibited a statistically significant difference compared with the control group (Fig. 4E). Overall, weekly NELL-PEG injection via the IP route significantly improved a number of bone architectural properties at the distal femur and lumbar vertebrae, confirmed by significant increases in BMD, BV/TV, and trabecular bone parameters at the end of the treatment.

### Bone remodeling activity by histology and immunohistochemistry

**FIG. 5.**
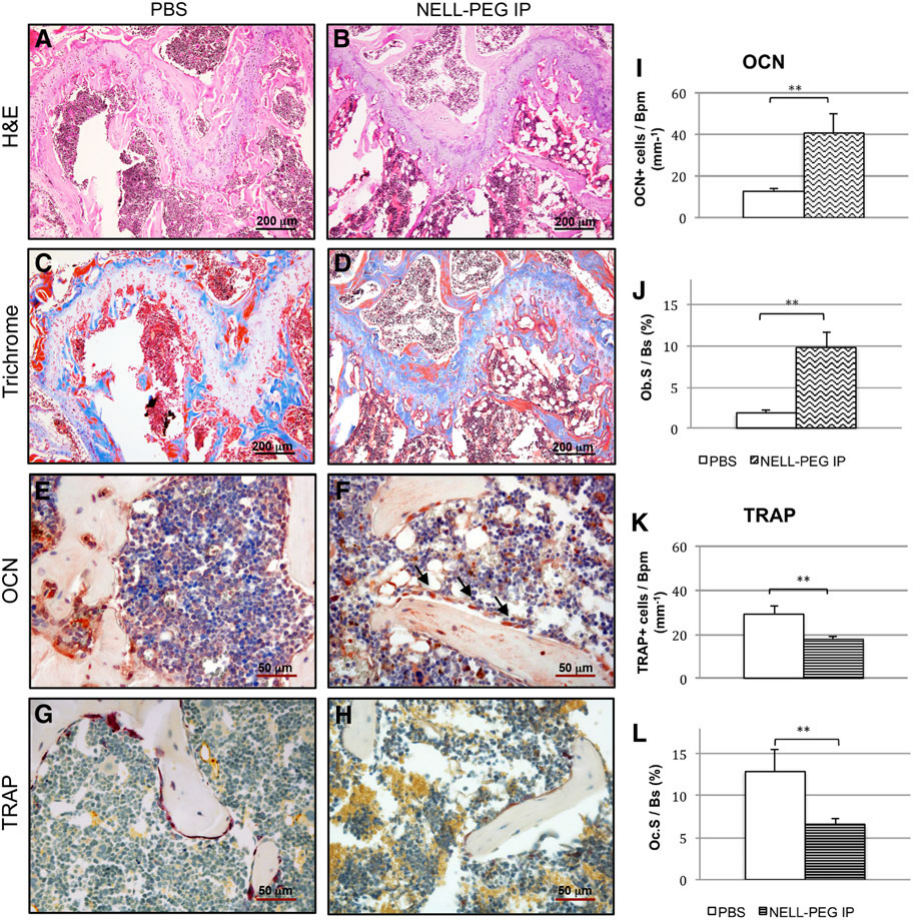
Histological analyses of distal femoral metaphysis in NELL-PEG-treated group and control. **(A, B)** H&E staining (bar, 200 μm) and **(C, D)** trichrome staining (bar, 200 μm) at the growth plate region show more trabecular bone formation in the NELL-PEG group compared with the PBS control. **(E, F)** High power view of OCN staining (bar, 50 μm) shows more osteoblasts in treated group (shown with arrows). **(G, H)** TRAP staining (bar, 50 μm) reveals more osteoclasts in the control group compared with the NELL-PEG injection group. **(I–L)** Quantification of bone remodeling process parameters that include osteocalcin+bone-lining cells per bone perimeter (OCN+cells/Bpm, mm^−1^) and surface (Ob.S/Bs, %), TRAP+bone-lining cells per bone perimeter (TRAP+cells/Bpm, mm^−1^), and surface (Oc.S/Bs, %). **Significant difference (*p* < 0.01) compared with the control group. Error bars represent standard error of the mean. H&E, hematoxylin and eosin; TRAP, tartrate-resistant acid phosphatase.

To assess the underlying cellular mechanisms of the bone remodeling process, histological analysis and static index assessment at the distal femoral metaphysis region were performed (Fig. 5). Consistent with the micro-CT findings, histological analysis exhibited a significant increase of trabecular bone formation in the NELL-PEG-treated group compared with the PBS control (Fig. 5A, B). Low-magnification view of the distal femoral metaphysis region stained with Masson's trichrome showed osteoid matrix (dark blue) ossifying into mature trabecular bone (red) and revealed actively remodeling bone in the NELL-PEG-treated group (Fig. 5C, D). Accordingly, results from OCN staining exhibited greater active bone formation (Fig. 5E, F), aligning with the results from TRAP staining that exhibited less osteoclastic activity in the treated group relative to the control (Fig. 5G, H). The bone remodeling process parameters that include osteocalcin+bone-lining cells per bone perimeter (OCN+cells/Bpm, mm^−1^) and surface (Ob.S/Bs, %), TRAP+bone-lining cells per bone perimeter (TRAP+cells/Bpm, mm^−1^), and surface (Oc.S/Bs, %) demonstrated that the NELL-PEG-treated group had a higher number of bone remodeling process indices compared with the control.

## Discussion

We have previously demonstrated that PEGylation of NELL-1 osteogenic protein significantly enhances NELL-1 pharmacokinetics by increasing the serum half-life, allowing the protein to be administered weekly IV to treat osteoporosis in mice.^10,11^ The present study revealed that a weekly injection of a double-dose NELL-PEG via IP injection also successfully increases BMD and trabecular bone formation with reduced bone resorption, presenting the IP administration is an alternative and more patient-friendly approach to systemically deliver NELL-PEG for the treatment for osteoporosis.

While many studies show the efficacy of various drug administration routes for drug delivery in mice, very few compare the effects of different administration routes on bone tissue regeneration. In this study, we compared the IP and SC administration of NELL-PEG. In the laboratory setting, IV, IP, and SC routes constitute the most frequently used drug delivery methods.^42^ While IV administration allows rapid dispersal of the drug into the circulatory system, the method is quite challenging to manipulate and entails noteworthy risks of inflammation, thrombophlebitis of the vein, and necrosis of the surrounding tissues. IP administration, however, is less invasive than IV administration and facilitates absorption due to the large surface area of the abdominal cavity and abundant blood supply at the injection site. Conversely, SC administration has the slowest rate of absorption. Compared with the IV route, the IP and SC routes are more favorable for drug delivery because they are not only less invasive but also allow for a greater volume of injection that serves as a slow-release and long-acting deposit of the drug.^42,44^ For long-term drug delivery formulations in humans, factors, such as the drug safety profile, ease of administration, subject accessibility and mobility, target area and injection site, and cost of therapy, should be taken into consideration. Nonetheless, in laboratory settings, the IP route has been more widely used due to its induction of greater drug effects in a shorter period.^42–45^

In the biodistribution study, in comparison to the SC administration group, the IP group showed greater NELL-PEG signal intensities in the liver, fat, and ovary, suggesting that the protein was absorbed and metabolized via the IP route but not via the SC route (Fig. 1). This observation may be attributable to the high molecular weight of NELL-PEG (863.1 kDa), which hinders diffusion into the capillaries near the injection site and subsequent distribution by the systemic circulation. To observe the efficacy and distribution of NELL-PEG uptake via the IP route, the dose was doubled (2.5 mg/kg), and imaging was conducted at two different time points (48 and 72 h) postinjection. The results (Fig. 1) suggest that injection of NELL-PEG via the IP route can facilitate the slow absorption of the protein from the injection site, thus maintaining a high-level protein concentration for a longer period. Taken together with our previous data, these findings led us to further examine the applicability of NELL-PEG injection via the IP route using an *in vivo* mouse model.

Our *in vivo* data demonstrate that not only a weekly IP double-dose (2.5 mg/kg) administration of NELL-PEG successfully increases BMD in the targeted bone sites (Fig. 2) but also the findings from the live micro-PET/CT scan using a radiolabeled tracer of ^18^F-NaF suggest that there is an active bone remodeling process that occurred at the targeted bone sites after NELL-PEG IP administration (Fig. 3). Tracer uptake of ^18^F-NaF corresponds to the bone turnover process that involves osteoblastic and osteoclastic activities on the bone surface, which concomitantly reflects the bone remodeling process.^46–49^ Consistently, micro-CT data showed that weekly NELL-PEG administration via the IP route promotes dramatic improvement in bone quality at the long bone sites by significantly escalating BMD and inducing robust trabecular bone formation (Fig. 4). The results from the histological analyses were consistent with the aforementioned findings that there was a significant increase in bone formation due to an increase in osteoblastic activity and reduced osteoclast-induced bone resorption (Fig. 5). As a whole, these findings indicate that weekly administration of NELL-PEG via the IP route significantly improves the bone properties at multiple skeletal sites.

## Conclusion

In summary, our results indicate that NELL-PEG injection via IP administration results in a greater protein uptake compared to SC administration and that weekly IP administration of a double-dose NELL-PEG successfully enhances BMD, relative volume of calcified tissue, and osteoblastic activity while reducing osteoclast activity in the targeted bone sites. Altogether, these findings suggest that IP administration is an excellent, patient-friendly alternative method to systemically deliver NELL-PEG for osteoporosis therapy.

## Abbreviations Used

AMIDE: A Medical Image Data Examiner
BMD: bone mineral density
BMSCs: bone mesenchymal stem cells
BV/TV: bone volume fraction
DXA: dual-energy X-ray absorptiometry
FDA: Food and Drug Administration
H&E: hematoxylin and eosin
IP: intraperitoneal
IV: intravenous
micro-CT: micro-computed tomography
micro-PET: micro-positron emission tomography
NELL-1: NEL-like molecule-1
NELL-PEG: PEGylation of NELL-1
OCN: osteocalcin
PBS: phosphate-buffered saline
PEG: polyethylene glycol
PFA: paraformaldehyde
SC: subcutaneous
TRAP: tartrate-resistant acid phosphatase
